# The effect of mechanical and chemo-mechanical temporary cement cleaning methods on shear bond strength with self-adhesive resin cement (an in-vitro study)

**DOI:** 10.1186/s12903-022-02672-7

**Published:** 2022-12-28

**Authors:** Ahmed Mohamed Arafa, Emad Aboalazm, Mostafa Hussein Kamel

**Affiliations:** 1 Department of Fixed Prosthodontics, Faculty of Dentistry, Beni-Suef University, Beni-Suef, Egypt; 2 Department of Restorative Dentistry, Faculty of Dentistry, Egyptian Russian University, Cairo, Egypt; 3 Department of Fixed Prosthodontics, Faculty of Dentistry, Misr International University, Cairo, Egypt

**Keywords:** Temporary cement, Mechanical cleaning, Chemo-mechanical cleaning, Adhesive resin cement, Shear bond strength

## Abstract

**Background:**

Adhesive tooth-colored restorations are strongly dependent on the substrate surface cleanliness to allow intimate contact between resin cement and dentin surface, so several methods were adopted for the total cleaning of temporary cement residues. This study aimed to assess the effect of mechanical and chemo-mechanical cleaning methods of temporary cement on the immediate shear bond strength of self-adhesive resin cement to dentin surface.

**Methods:**

Forty freshly extracted lower first premolars were cut to expose a flat dentin surface. Discs of temporary crown composite resin material were constructed and cemented to the flat dentin surface using resin-based and non-eugenol temporary cement then stored at room temperature in distilled water. Dividing of samples into two groups according to the method of temporary cement cleaning. Group I (n = 20) mechanical cleaning using the rotary instrument, and group II (n = 20) chemo-mechanical cleaning using chlorhexidine-containing scrub. CAD/CAM reinforced Composite discs were bonded to the dentin surface using self-adhesive composite resin cement, then measurement of shear bond strength was done using a universal testing machine. Further analysis of failure mode after debonding was performed by Scanning electron microscope.

**Results:**

No statistically significant difference was found between the mean shear bond strength of the two cleaning methods (*P*-value = 0.636). Regardless of the cleaning method, the group cemented with resin-based temporary cement showed statistically significantly higher mean shear bond strength than non-eugenol temporary cement (*P*-value = 0.048).

**Conclusion:**

Both cleaning methods (mechanical and chemo-mechanical) applied in this study were effective in cleaning temporary cement remnants from the dentin substrate surface with statistically significant differences between results of shear bond strength with significantly higher values recorded with resin-based temporary cement.

## Background

Successful tooth-colored restorations are strongly correlated to the nature and strength of bonding between the restorative material and tooth substrate, which determines the longevity of true adhesive junction [1, 2]. Studies have emphasized that the temporization phase, which is mandatory for the protection of tooth structure until the fabrication of the final restoration, had a negative impact on bond strength between resin cement and dentin substrate [3]. Remnants of the temporary cement (macroscopic or microscopic) can prevent proper contact between the adhesive systems and dentin which in turn may adversely affect bonding with tooth structure, especially that eugenol-containing temporary cements that are widely used for their advantages of being bacteriostatic effect, effective dentin sealing abilities and sedative effect on the vital pulp tissue [4]. This strong durable bond between dentin substrate and final indirect ceramic restorations is required to obtain better marginal adaptation, which can help to prevent marginal gap and microleakage and improve the prognosis of teeth restored with indirect restorations [5, 6]. These eugenol-containing temporary cements have detrimental effects on bonding as they interfere with surface wettability and inhibit the polymerization reaction of resin cement. Studies have emphasized the impact of total removal of temporary cement on the enhancement of bond strength and protection of dental and gingival structure by reducing microleakage harmful effects [7, 8]. These disadvantages led to the introduction of other eugenol-free types and resin-based temporary cement, which can be easily removed and would not affect the polymerization of resin cements [9]. Also, smear layer existence is another factor that can influence the adequate bonding with final restorations. The conventional resin cement and dentin bonding agent had been included multistep procedure making it a technique sensitive, time-consuming, susceptible postoperative hypersensitivity and hydrolytic degradation. A combination of both tooth structure pre-treatment and resin infiltration in a one-step would overcome some limitations with a multistep technique [5]. This led to self-adhesive resin cement introduction to remove the need for further etching, priming, or bonding steps that allow clinicians to use a cementation protocol through single clinical step. This cement provides micromechanical retention resulting from demineralization and infiltration of the dental tissues due to incorporated multifunctional phosphoric acid methacrylates [5].

Irrespective of the resin cement type used, provisional cement remnants can adversely influence the bonding strength with dental tissues, so several methods were adopted for total cleaning of temporary cement residues including mechanical and chemical methods [9–11]. The chemical methods utilize various cleaning agents containing ethyl acetate, acetone, ethanol, or chlorhexidine digluconate [12, 13]. Other mechanical methods were also found to be effective for this purpose, including just the application of a rotational brush with water coolant [14]. Chlorhexidine (CHX) which is used as an antimicrobial agent in the oral cavity, has been stated that it can stop the action of Matrix metalloproteinases (MMPs), which can cause degradation of all extracellular matrix components, enhancing the bond longevity between dentin substrate and different adhesives [15, 16].

This study aimed was to assess the effect of recent methods of mechanical or mechanical combined with chemical methods for total cleaning of temporary cement residues and their impact on immediate shear bond strength (SBS) with dentin, where a gap of knowledge exists on the effect of combining mechanical with chemical methods of temporary cement cleaning on shear bond strength. The null hypothesis was that there was no significant difference in shear bond strength with both cleaning methods used to remove remnants of temporary cement material.

## Methods

The sample size was calculated depending on a previous study [14]. According to this study, the minimally accepted sample size was 10 per group, when the response within each subject group was normally distributed with a standard deviation of 1.47, the true mean difference was 1.95 when the power was 80% & type I error probability was 0.05.

The materials used in this study are mentioned in (Table 1).

**Table 1:** Materials and equipment used

Material	Product name	Manufacturer	Composition
1. Temporary Non- Eugenol Cement	NETC Dental Paste-paste dual syringe type	Meta Biomed CO LTD, Korea	Base: Zinc Oxide, Mineral oil Catalyst: Rosin, Nonanoic acid
2. Temporary resin-based cement	Provitemp	Itena Clinical products, France	Fluoride—Chlorhexidine—Potassium Nitrate—Methacrylates—Urethan dymethacrylate—Polymerisation activator
3. Chlorhexidine Antibacterial slurry	Consepsis Scrub	Ultradent Products Inc., USA	2.0% chlorhexidine gluconate (disinfecting abrasive scrub
4. Coronal brush	STARbrush	Ultradent Products Inc., USA	
5. Rotary cleaning instrument	OptiClean	KerrHawe, Switzerland	Tool with flexible coating & 1.65 mm tip diameter. The optimized abrasive particles within a silicone matrix
6. Reinforced CAD/CAM composite block	Brilliant Crios	Coltene, Switzerland	Innovative submicron hybrid composite material 1. Dental glass: Barium glass (Size < 1.0 µm) 2. Amorphous silica: SiO_2_ (Size < 20 nm) 3. Resin matrix: Cross-linked methacrylates 4. Pigments: Inorganic pigments such as ferrous oxide or titanium dioxide
7. Resin cement	SoloCem	Coltene, Switzerland	Self-adhesive dual-cured radiopaque composite-resin cement Methacrylates, Zinc oxide, Dental glass
8. Bonding agent	One coat 7 universal	Coltene, Switzerland	One-component light-cured self-etching nanofilled adhesive with acetone-based solvents
9. Temporary crowns & bridge material	Cool Temp Natural	Coltene, Switzerland	Bis-acryl composite (Methacrylates, Bariumglass silanized, Amorphous silica hydrophobed)

### Teeth specimen preparation

Forty freshly extracted lower first premolars for orthodontic reasons (All procedures were performed by the declaration of Helsinki, with the approval of the Research Ethics Committee (FDBSU-REC) of Faculty of Dentistry, Beni-Suef University, Egypt (Approval number: # REC-FDBSU/04082022–02/AA). Extracted lower first premolars for orthodontic reasons that would otherwise be discarded following extractions as part of routine patient care were collected. They were selected free from caries, and abrasion cavities and had intact crowns. The extracted teeth were scaled to remove organic debris and then stored in 0.9% saline (ADWIC, Pharmaceutical division, Abs Zabal, Egypt) until use to prevent desiccation during storage. Each tooth specimen was vertically mounted in self-cure polymethyl-methacrylate (PMMA) resin blocks (Acrostone, Egypt) along its long axis by using a specially designed split brass mold machine milled to standardize the fabrication of the acrylic blocks. A paralleling device (BEGO, Germany) was used to ensure the centralization and alignment of the tooth specimen to the mold at a predetermined depth till complete polymerization, then stored again in 0.9% saline. The teeth specimens mounted in resin blocks were then cut horizontally using an electrical high-precision saw (Isomet 4000, microsaw, Buehler Ltd, USA) with a diamond cutting disc under copious water cooling to get a flat coronal dentin surface.

Random division of teeth specimens into two groups (n = 20) was done according to the cleaning method used to remove temporary cement from the dentin surface. Group I: (Mechanical cleaning) (OptiClean, Kerr Hawe, Switzerland) utilized the mechanical cleaning method and Group II: (Chemo-mechanical cleaning) (Consepsis Scrub, Ultradent Products Inc., USA) utilized mechanical combined with a chemical cleaning agent. Each one was then subdivided into two subgroups (n = 10) according to the type of temporary cement used, subgroup R: Resin-based temporary cement and subgroup Non-E: non-eugenol temporary cement. (Table 2).

**Table 2:** Showing specimens grouping

	Group I (Mechanical cleaning) OptiClean	Group II (Chemo-mechanical cleaning) Consepsis Scrub
*Subgroup R* (Resin-based temporary cement)	10	10
*Subgroup Non-E* (Non-Eugenol temporary cement)	10	10

### Temporary restoration preparation

To simulate the clinical situation, forty discs of temporary composite resin (Cool Temp Natural, Coltene, Switzerland) with a diameter of 3.5 mm and a thickness of 1.5 mm were constructed and cemented to the flat dentin surface with the tested temporary cement under a 2.5 kg static load for 5 min. Twenty discs were cemented using resin-based temporary cement extruded from a syringe with auto-mixing tip for subgroup R; the other twenty were cemented using non-eugenol temporary cement for subgroup Non-E, which was extruded from a syringe with auto-mixing tip. Storage of teeth specimens with cemented temporary composite resin discs in distilled water at room temperature [17] for five days was done after the complete setting of temporary cement (3–4 min for Provytemp resin-based temporary cement, 6 min for NETC Non-eugenol temporary cement). After this storage period, the temporary composite resin discs were removed manually using a large excavator, and temporary cement was cleaned with a scaler till obtaining macroscopically clean dentin surfaces, then were thoroughly rinsed with water. then, all teeth specimens were subjected to tested cleaning procedures as follows; for group I: (mechanical cleaning), the dentin surface of teeth specimens was cleaned using a rotary instrument (OptiClean, KerrHawe, Switzerland) with a handpiece for 15 s at 5000 rpm with water cooling. For group II: (Chemo-mechanical cleaning), the dentin surface of teeth specimens was cleaned using Consepsis Scrub and StarBrush at a low speed for 15 s.

### Reinforced CAD/CAM composite disc preparation

Composite discs simulating final indirect CAD/CAM restoration were grounded using a lathe to obtain rods from reinforced CAD/CAM composite block (Brilliant Crios, Coltene, Switzerland) then, discs were made by slicing the rods utilizing an electrical high-precision saw (Isomet 4000, microsaw, Buehler Ltd, USA) with diamond cutting disc under copious water cooling to get a flat surface disc-shaped specimens with a diameter of 3.5 mm and a thickness of 1.5 mm. The surfaces intended for adhesive bonding were polished with standard composite polishers (medium, fine and extra fine diamonds).

### Adhesive bonding procedures

The dentin surface of teeth specimens was dried by oil-free air blowing followed by a bonding agent (One coat 7 universal, Coltene, Switzerland) applied to the dentin surface by the disposable dental brush and was rubbed well for 20 s, then gently air dried for 5 s followed by light curing for 10 s. Sandblasting of reinforced CAD/CAM composite discs was done using corundum (25–50 μm Aluminium oxide at 1.5 bar) following the manufacturer's instructions followed by bonding agent application. The reinforced CAD/CAM composite discs were bonded to the dentin surface using self-adhesive composite resin cement (SoloCem, Coltene, Switzerland) using an auto-mixing tip and following the manufacturer's instructions. By applying light pressure, the discs were seated in place, uncured excess cement was removed with a spatula, and finer excess removal was completed after brief polymerization for 3 s. The complete chemical setting of cement was done under a static load of 5 kg while holding the restoration in position. Each specimen was light cured at a 1 mm distance for 40 s using a visible light-curing unit (Woodpecker LED-D Wireless, Mident Industrial Co., Ltd. Henan, China) with an output power of 850–1000 mW/cm^2^. Finally, teeth specimens were stored in distilled water at room temperature until the testing procedure.

### Shear bonding testing procedure

It was done using a universal testing machine (Instron 3345, Instron Corporation, England). Each specimen was mounted onto a metal holder in the universal testing machine equipped with a 1-kN load cell at a crosshead speed of 1 mm/min. The tightened specimen was stabilized until the knife-edge chisel centralized as close to the reinforced composite disc-tooth junction as possible. The ultimate shear load to failure was recorded in newtons (N). Using machine software (BlueHill 3, Instron, England), the maximum ultimate load to failure (N) was divided by the bonded cross-sectional area (mm^2^) to get the average bond strength (MPa). The means and standard deviations were recorded.

### Failure mode analysis

After finishing the testing procedure, careful examination for captured images of the bonded surfaces of reinforced composite disc and dentin surface from each specimen was performed utilizing a digital microscope (Dino-Lite Pro, Olympus, Tokyo, Japan). Identification of the failure mode was made as follows: adhesive tooth/cement (no remnants of resin cement left on the dentin surface), cohesive in cement (the fracture located in the cement) and mixed (remnants of resin cement partially left on the dentin surface with dentin surface exposed).

### Scanning electron microscopy (SEM)

Subjective assessment of the dentin surface properties to assess the effect of the different cleansing methods was done utilizing a scanning electron microscope (SEM) (Quanta FEG 250, FEI Company, Hillsboro, Oregon-USA). Images of dentin were recorded before and after each cleaning procedure. After mounting the samples onto SEM stubs, SEM conditions were a 10.1 mm working distance, with an in-lens detector with an excitation voltage of 20 kV. The tested dentin surface representing each type of failure was also evaluated after shear bond testing.

### Statistical analysis

Numerical data were explored for normality by checking the distribution of data and using tests of normality (Kolmogorov–Smirnov and Shapiro–Wilk tests). All data revealed a normal (parametric) distribution. Data were presented as mean and standard deviation (SD) values. Assessment of the effect of cleaning method, cement type and their interaction on mean shear bond strength was done by two-way Analysis of Variance (ANOVA). Bonferroni’s post-hoc test was used for pair-wise comparisons when ANOVA test was significant. The significance level was set at *P* ≤ 0.05. Statistical analysis was performed with IBM SPSS Statistics for Windows, Version 23.0. Armonk, NY:IBM Corp.

## Results

### Two-way ANOVA results

The results revealed that the cleaning method (regardless of cement type) had no statistically significant effect on mean shear bond strength (*P*-value = 0.636). Cement type (regardless of cleaning method) had a statistically significant effect on mean shear bond strength (*P*-value = 0.048). The interaction between the two variables had no statistically significant effect on mean shear bond strength (*P*-value = 0.848). Since the interaction between the variables is non-statistically significant, so the variables are independent of each other.

#### Effect of cleaning method

**Fig. 1:**
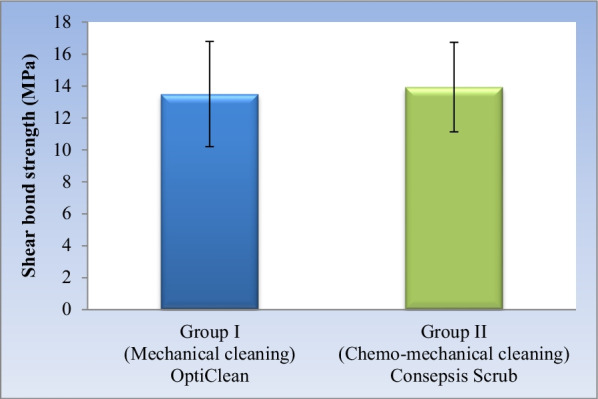
Bar chart showing mean and standard deviation values for shear bond strength of the two cleaning methods regardless of cement type

Regardless of cement type, no statistically significant difference was found between the mean shear bond strength of the two cleaning methods (*P*-value = 0.636). (Table 3, Fig. 1).

**Table 3:** The mean, standard deviation (SD) values and results of two-way ANOVA test for comparison between shear bond strength (MPa) of the two cleaning methods regardless of cement type

Group I (Mechanical cleaning) OptiClean		Group II (Chemo-mechanical cleaning) Consepsis Scrub		*P*-value
Mean	SD	Mean	SD	
13.5	3.3	13.94	2.81	0.636*

^*^ Significant at *P* ≤ 0.05

#### Effect of cement type

**Fig. 2:**
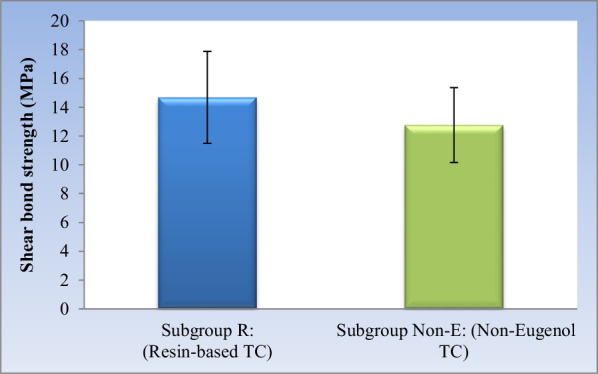
Bar chart showing mean and standard deviation values for shear bond strength of the two cement types regardless of cleaning method

Regardless of the cleaning method, resin-based temporary cement showed statistically significantly higher mean shear bond strength than non-eugenol temporary cement (*P*-value = 0.048). (Table 4, Fig. 2).

**Table 4:** The mean, standard deviation (SD) values and results of two-way ANOVA test for comparison between shear bond strength (MPa) of the two cement types regardless of cleaning method

Subgroup R: (Resin-based TC)		Subgroup Non-E: (Non-Eugenol TC)		*P*-value
Mean	SD	Mean	SD	
14.68	3.19	12.76	2.6	0.048*

^*^ Significant at *P* ≤ 0.05

#### Effect of different interactions on shear bond strength

##### Comparison between cleaning methods

**Fig. 3:**
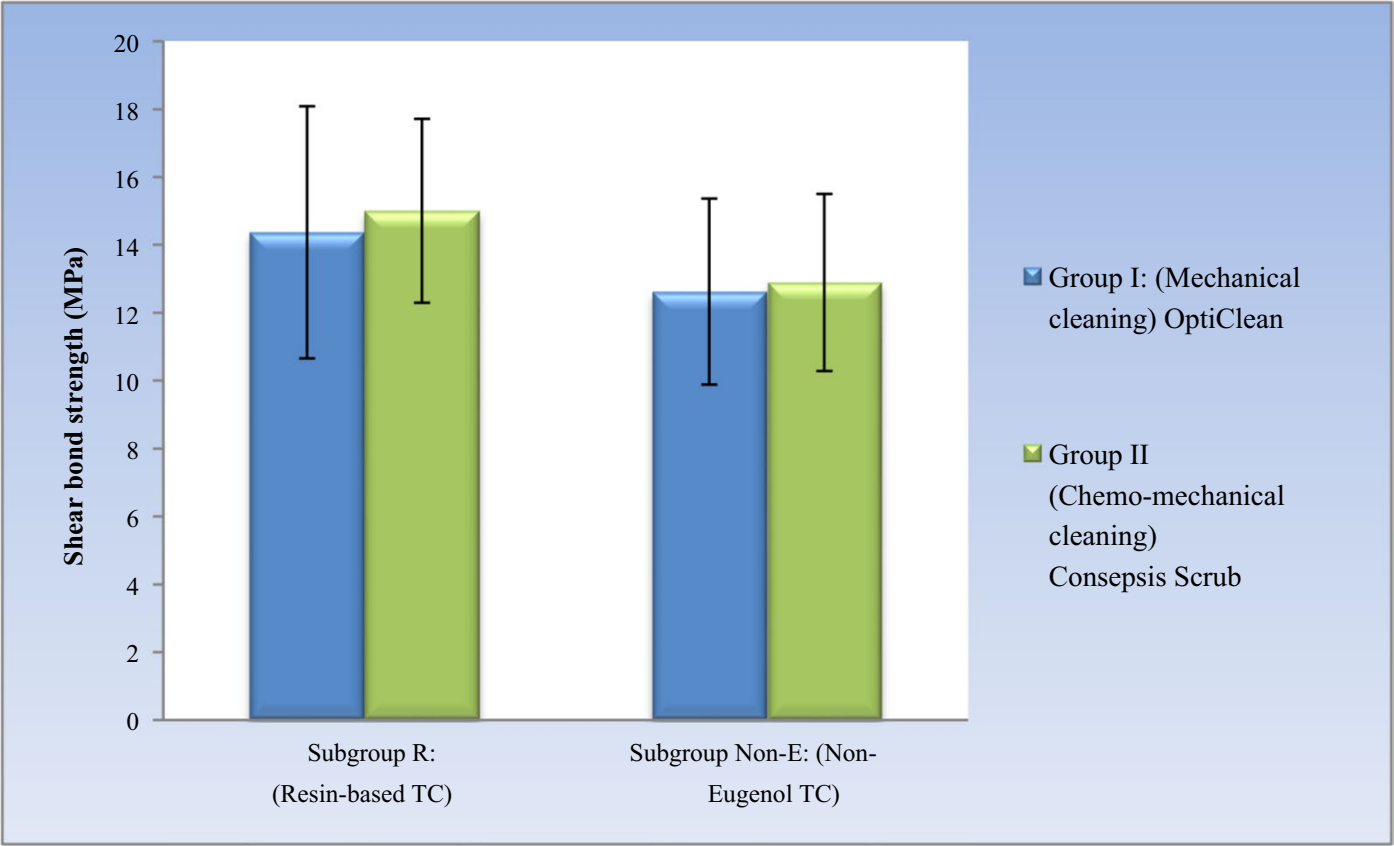
Bar chart showing mean and standard deviation values for shear bond strength of different interactions of variables

Whether with resin-based temporary cement or non-eugenol temporary cement, no statistically significant difference was found between the mean shear bond strength of the two cleaning methods (*P*-value = 0.639) and (*P*-value = 0.842), respectively. (Table 5, Fig. 3).

**Table 5:** The mean, standard deviation (SD) values and results of two-way ANOVA test for comparison between shear bond strength (MPa) of different interactions of variables

Cement type	Group I (Mechanical cleaning) OptiClean		Group II (Chemo-mechanical) Consepsis Scrub		*P*-value
	Mean	SD	Mean	SD	
*Subgroup R* (Resin-based temporary cement)	14.37	3.72	15	2.71	0.639*
*Subgroup Non-E* (Non-Eugenol temporary cement)	12.62	2.74	12.89	2.61	0.842*
*P*-value	0.198		0.122		

^*^ Significant at *P* ≤ 0.05

##### Comparison between cement types

Whether with mechanical cleaning (Opticlean) or chemo-mechanical cleaning (Consepsis Scrub), no statistically significant difference was found between the mean shear bond strength of the two cement types (*P*-value = 0.198) and (*P*-value = 0.122), respectively. (Table 5, Fig. 3).

**Fig. 4:**
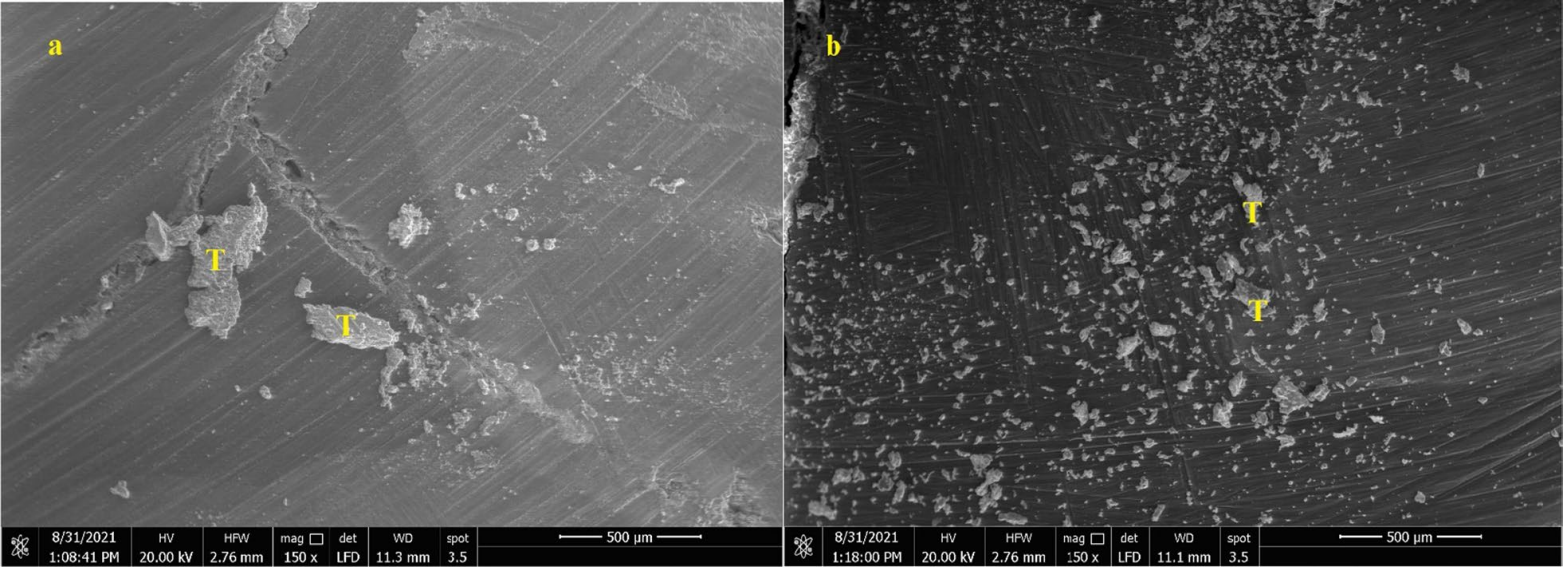
SEM micrograph of dentin surface before cleaning of temporary cement (200 X) **a** Non-Eugenol TC, **b** Resin-based TC, T: Remnants of temporary cement

**Fig. 5:**
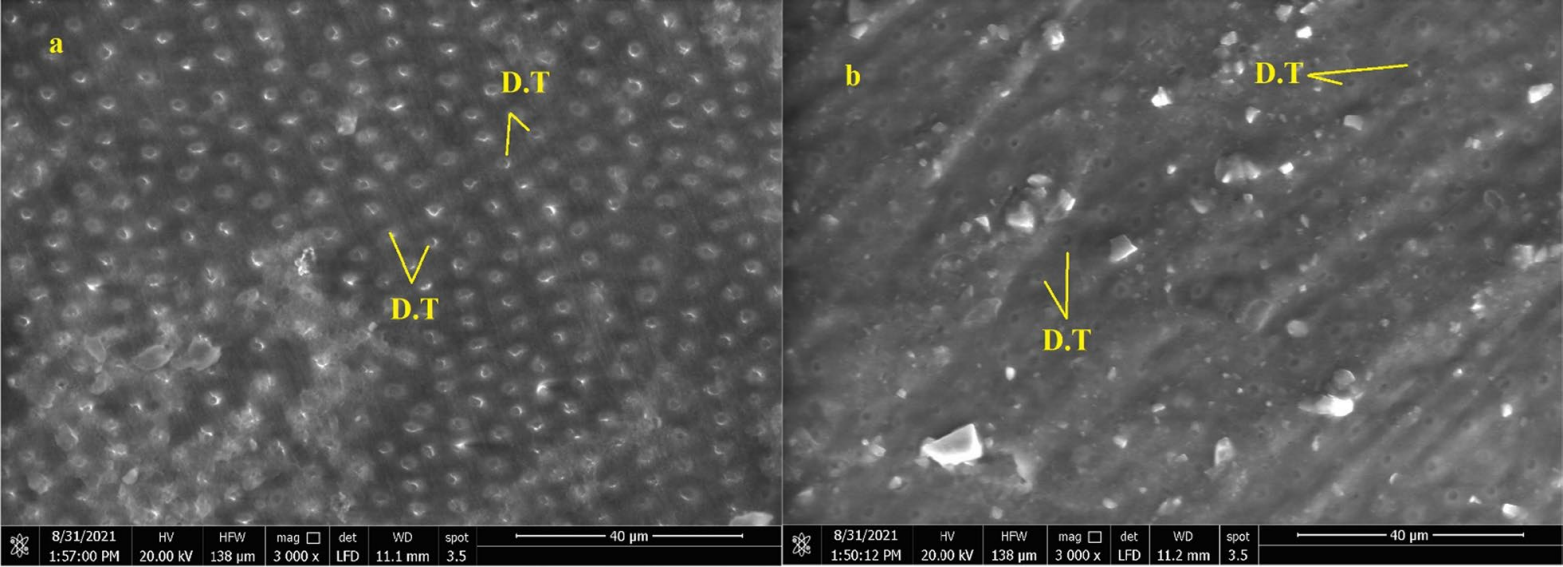
SEM micrograph of dentin surface after cleaning of Non-Eugenol temporary cement (3000 X) **a** after mechanical cleaning procedure (OptiClean), **b** after chemo-mechanical cleaning procedure (Consepsis Scrub), D.T: open dentinal tubules

**Fig. 6:**
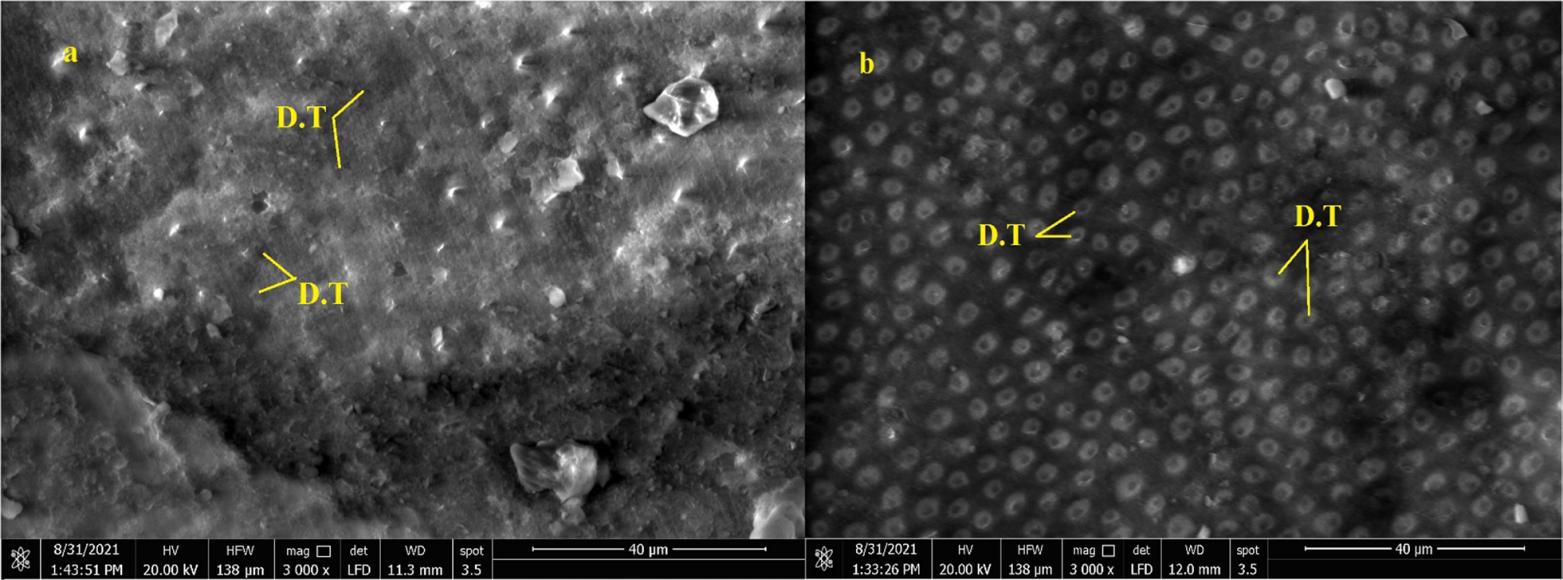
SEM micrograph of dentin surface after cleaning of Resin-based temporary cement (3000 X) **a** after mechanical cleaning procedure (OptiClean), **b** after chemo-mechanical cleaning procedure (Consepsis Scrub), D.T: open dentinal tubules

Images of dentin were recorded before and after each cleaning procedure (Figs. 4a, b, 5a, b and 6 a, b).

**Fig. 7:**
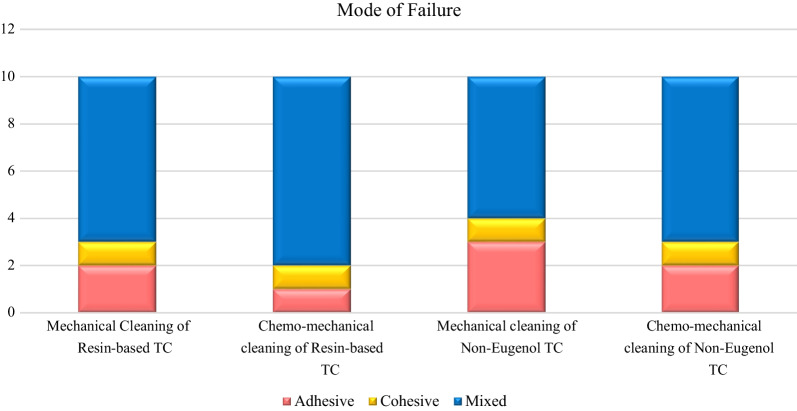
Bar chart showing mode of failure analysis of all groups

**Fig. 8:**
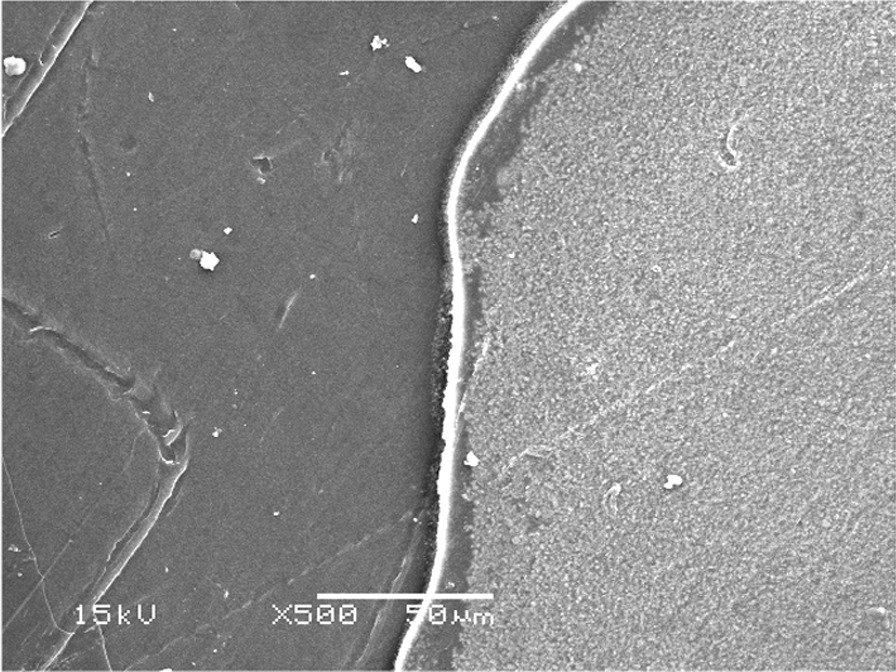
Topographical analysis of the bonding dentine surface showing mixed mode of failure of cement layer (500X)

**Fig. 9:**
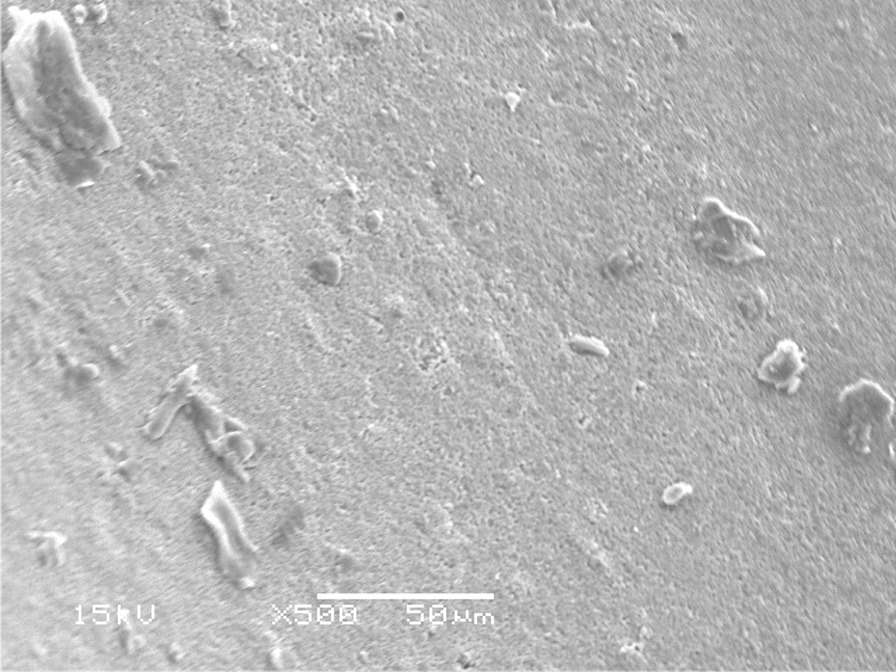
Topographical analysis of the bonding dentine surface showing adhesive mode of failure of cement layer (500X)

**Fig. 10:**
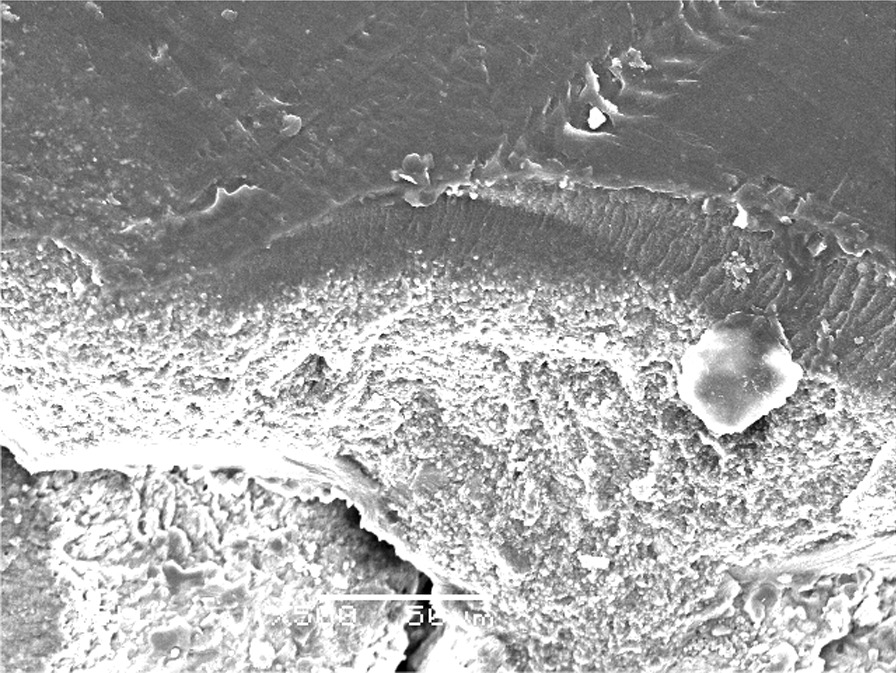
Topographical analysis of the bonding dentine surface showing cohesive mode of failure within cement layer (500X)

The failure analysis showed that the most common failure mode was the mixed type, followed by adhesive type and the least common was the cohesive type. (Fig. 7) SEM evaluations of surface topography at 500 X were used to study the failure modes of different groups. (Figs. 8, 9 and 10).

## Discussion

Temporization is a vital phase in restorative dentistry, as protection of tooth structure till the fabrication of the final restoration reduces complications on tooth structure. Yet studies have emphasized the role of temporary cement residues in the prevention of proper bonding with dentin substrate [3]. This led to the development of multiple methods, either chemical, mechanical or chemo-mechanical, to clean the dentin substrate surface prior to the bonding procedure. Studies have emphasized the effect of application of different cleaning procedures to dentin surface not only removed the temporary cement but has shown different effects on the smear layer ranging from simple removal of contaminants such as debris and blood to partial or total removal of the smear layer promoting interaction between the resin cement and the collagen network on the dentin surface [5].

In this study, two cleaning methods were evaluated, including mechanical (OptiClean) and chemo-mechanical (Consepsis Scrub) containing abrasive particles in combination with Chlorhexidine. The selection of eugenol-free temporary cement was preferred to avoid the adverse effect of eugenol on the shear bond strength [7, 8]. Two types of eugenol-free temporary cement were applied to dentin sections, resin-based temporary cement (Provytemp) and Non-Eugenol temporary cement (NETC). Selection of self-adhesive bonding agent and resin cement to bond with dentin was selected due to its benefits, including simplified procedures, reduced postoperative hypersensitivity and durable bond with dentin [18, 19]. Although several suggested methods for cleaning temporary cement, some utilize mechanical methods, while others used chemical agents. The chemical agents affected only the superficial layer of the dentin so that further mechanical cleansing procedures might be needed. Mechanical cleansing procedures involve rotary instrumentation with pumice, an air polisher or a micro-particle abrasion system. The rotary instrumentation with pumice or burs was recommended by many authors [2, 14]. The mechanical methods of temporary cement removal were considered effective due to their capability to remove the smear layer totally or partially, which can help dentin hybridization with resin cement [14]. The application of pumice to enhance bond strength revealed variable results. Some investigators encourage the use of the pumice for removal of temporary cement [20, 21] as removal of temporary cement helps to improve the shear bond strength and protects the dental and gingival structure by reducing microleakage [22], while other studies have reported opposing results [23, 24].

The results of this study revealed statistically non-significant differences in shear bond strength between both cleaning methods used (*P*-value = 0.636). This finding supports the efficacy of the mechanical cleaning method and the chemo-mechanical method [25]. Both methods don’t only effectively remove the remnants of temporary cement, it also removes partially or totally the smear layer [14]. This is due to the Consepsis paste containing abrasive particles, which augment the effect of CHX. The findings in the present study were in agreement with data from a previous study by de Castro et al. [26], which revealed no harmful effect of CHX on the bond strength. This may be attributed to the CHX effect in increasing the surface energy of the tooth surface and improving dentin wettability with adhesive.

Another study stated that using CHX was ineffective for cleaning provisional cement off dentin surface prior to application of self-adhesive resin cement [14]. The resulting low shear bond strength values were attributed to the ineffective removal of the smear layer by using CHX solution. Also, due to its affinity for phosphate groups (cationic properties), this interferes with the acidic monomers of self-adhesive resin cement affecting the bonding with loose apatite within the smear layer [27].

Despite the antimicrobial effect of Chlorhexidine gluconate present in Consepsis paste on Matrix Metalloproteinases (MMPs), it didn’t possess further confirmed effect on the immediate shear bond strength values [28–32]. Studies described the Chlorhexidine effect on Matrix Metalloproteinases (MMPs) enzymes, thus helping in maintenance and stability of the hybrid layer with and providing long-term successful bonding with dentin [15, 16, 31, 33].

In subgroups where resin-based temporary cement (Provytemp) was applied, the results of shear bond strength were statistically significantly higher than other subgroups where Non-Eugenol temporary cement (NETC) was applied (*P*-value = 0.048); these results are coincident with other studies that attributed this to the ease of removal of this type of temporary cement without leaving remnants [9, 34, 35]. The results of our study were confirmed by SEM photomicrographs of the dentin surface before the application of any cleaning method; the remnants of temporary cement were markedly seen adherent to the dentin surface. Further photomicrographs after the application of cleaning methods have shown its effect not just on the removal of the adherent remnants but also on the exposure of the dentinal tubules, indicating the removal of the smear layer. The failure analysis after debonding of the specimens has shown that mixed type of failure was the most common mode indicating effective bonding with dentin substrate.

A limitation of this study was the lack of simulation of oral conditions on shear bond strength stability following the application of temporary cement cleaning methods, including thermal fluctuation, which can give a further view on the effect of both studied cleaning methods on bond stability, referring to some of the controversy in the literature about the role chlorhexidine on the long-term bond stability.

The null hypothesis of this study was accepted as there was no significant difference in shear bond strength with both cleaning methods used to remove remnants of temporary cement material.

## Conclusions

Within the limitations of this study, the following was concluded:

1. Both cleaning methods applied in this study were efficient in the cleaning of temporary cement remnants from the dentin substrate surface.
2. Both cleaning methods applied to remove resin-based temporary cement revealed significantly higher values of shear bond strength.
3. The mechanical method of temporary cement cleaning was effective, and no further positive effect was gained from adding a chemical agent as chlorhexidine gluconate on immediate shear bond strength.

### Future studies

Further research is needed to evaluate the effect of thermocycling on shear bond strength after the application of both cleaning methods of temporary cement remnants from the dentin substrate surface.

## Data Availability

The datasets used and/or analysed during the current study are available from corresponding author on reasonable request due to privacy reasons and large data size.

## Abbreviations

CHX: Chlorhexidine
CAD/CAM: Computer-aided designing/Computer-aided manufacturing
SBS: Shear bond strength
SEM: Scanning electron microscopy
MMPs: Matrix metalloprotinases
